# Hospitalisation for COVID-19 predicts long lasting cerebrovascular impairment: A prospective observational cohort study

**DOI:** 10.1016/j.nicl.2022.103253

**Published:** 2022-11-07

**Authors:** Kamen A. Tsvetanov, Lennart R.B. Spindler, Emmanuel A. Stamatakis, Virginia F.J. Newcombe, Victoria C. Lupson, Doris A. Chatfield, Anne E. Manktelow, Joanne G. Outtrim, Anne Elmer, Nathalie Kingston, John R. Bradley, Edward T. Bullmore, James B. Rowe, David K. Menon, The Cambridge NeuroCOVID Group, The NIHR COVID-19 BioResource, The Cambridge NIHR Clinical Research Facility, The CITIID-NIHR BioResource COVID-19 Collaboration

**Affiliations:** a Department of Clinical Neurosciences, University of Cambridge, Cambridge, United Kingdom; b Department of Psychology, University of Cambridge, Cambridge, United Kingdom; c Division of Anaesthesia, Department of Medicine, University Cambridge, Cambridge, United Kingdom; d Wolfson Brain Imaging Centre, University of Cambridge, Cambridge, United Kingdom; e Cambridge Clinical Research Centre, NIHR Clinical Research Facility, Cambridge University Hospitals NHS Foundation Trust, Addenbrooke's Hospital, Cambridge, United Kingdom; f NIHR BioResource, Cambridge University Hospitals NHS Foundation, Cambridge Biomedical Campus, Cambridge, United Kingdom; g Department of Haematology, School of Clinical Medicine, University of Cambridge, Cambridge Biomedical Campus, Cambridge, United Kingdom; h Department of Medicine, University of Cambridge, Addenbrooke's Hospital, Cambridge, United Kingdom; i Department of Psychiatry, University of Cambridge, Cambridge Biomedical Campus, Cambridge, United Kingdom; j Medical Research Council Cognition and Brain Sciences Unit, Department of Psychiatry, Cambridge, United Kingdom

**Keywords:** Cerebrovascular, Microvascular, Cardiorespiratory, Neurology, COVID-19, SARS-CoV-2

## Abstract

Human coronavirus disease 2019 (COVID-19) due to severe acute respiratory syndrome coronavirus-2 (SARS-CoV-2) has multiple neurological consequences, but its long-term effect on brain health is still uncertain. The cerebrovascular consequences of COVID-19 may also affect brain health. We studied the chronic effect of COVID-19 on cerebrovascular health, in relation to acute severity, adverse clinical outcomes and in contrast to control group data. Here we assess cerebrovascular health in 45 patients six months after hospitalisation for acute COVID-19 using the resting state fluctuation amplitudes (RSFA) from functional magnetic resonance imaging, in relation to disease severity and in contrast with 42 controls. Acute COVID-19 severity was indexed by COVID-19 WHO Progression Scale, inflammatory and coagulatory biomarkers. Chronic widespread changes in frontoparietal RSFA were related to the severity of the acute COVID-19 episode. This relationship was not explained by chronic cardiorespiratory dysfunction, age, or sex. The level of cerebrovascular dysfunction was associated with cognitive, mental, and physical health at follow-up. The principal findings were consistent across univariate and multivariate approaches. The results indicate chronic cerebrovascular impairment following severe acute COVID-19, with the potential for long-term consequences on cognitive function and mental wellbeing.

## Highlights

• Chronic cerebrovascular dysfunction (CVD) was related to the severity of acute COVID-19.
• This relationship was not explained by other COVID-19 risk factors or outcomes.
• CVD was associated with worse cognitive, mental, and physical health at follow-up.
• The physiological and genetic signature of this CVD shapes the composition of cell-types, metabolism and vasoreactivity essential for neuronal homeostasis.

## 1 Introduction

Severe acute respiratory syndrome coronavirus-2 (SARS-CoV-2) causes human coronavirus disease 2019 (COVID-19) with multi-system effects that include neurological, vascular and neurovascular injury. Acute neurological sequelae are common, ranging from mild dizziness, headaches and anosmia to severe encephalitis, stroke and delirium (Chen et al., 2021, Hensley et al., 2022, Paterson et al., 2020, Zubair et al., 2020). These sequelae may arise from systemic physiological insults (e.g. hypoxia, hypotension, dysautonomia), coagulation dysfunction, large vessel occlusion, arterial stiffness, impaired vasoreactivity, neurotropic infection, parenchymal haemorrhage, or autoimmune responses against diverse antigens (Chen et al., 2021, Marcic et al., 2021, Mohkhedkar et al., 2021, Schnaubelt et al., 2021). Acute COVID-19 has also been associated with microvascular injury from vasculitis or endotheliitis (McGonagle et al., 2021a, McGonagle et al., 2021b), with endotheliopathy (Kakarla et al., 2021), vasogenic oedema and microthrombosis in the acute phase (Iba et al., 2020, Levi et al., 2020) and hypoperfusion in the subacute phase (Hosp et al., 2021). While this acute pathophysiology is detectable using neuroimaging (Hanafi et al., 2020, Lersy et al., 2021, Newcombe et al., 2021), the persistence and effects of cerebrovascular dysfunction over the medium- and long-term remain unknown.

An important aspect of cerebrovascular function is the capacity of cerebral vessels to constrict or dilate in response to physiological conditions such as alterations in carbon dioxide (CO_2_) and oxygen tension. This cerebrovascular reactivity (Willie et al., 2014) regulates regional blood flow via pH-dependent modulation of vascular smooth muscle tone (Ainslie et al., 2005, Jensen et al., 1988, Lambertsen et al., 1961, Lassen, 1968), but is compromised by arterial stiffness, compromised endothelial function (Brandes et al., 2005), or disorders including hypertension, traumatic brain injury and dementia. Poor cerebrovascular reactivity may also increase the risk of neurodegeneration (Gao et al., 2013).

We therefore assessed the impact of COVID-19 on chronic cerebrovascular reactivity after hospitalisation. We used a well-established non-invasive imaging method, exploiting naturally occurring fluctuations in arterial CO_2_ induced by variations in cardiac and respiratory cycles, which moderate the blood oxygenation level-dependent (BOLD) signal underlying functional magnetic resonance imaging (Birn et al., 2006, Glover et al., 2007). The BOLD signal variability at rest, known as resting state fluctuation amplitudes (RSFA), is a safe, scalable and robust alternative to the gold standard approaches of measuring cerebrovascular reactivity with MRI (Golestani et al., 2016, Kannurpatti and Biswal, 2008, Liu et al., 2017, Tsvetanov et al., 2021b, Tsvetanov et al., 2021c, Tsvetanov et al., 2015). It is easier and safer to apply in clinical cohorts than experimental hypercapnia, breath-holding and drug interventions (Keyeux et al., 1995, Rostrup et al., 1996, Rostrup et al., 1994, Wagerle and Mishra, 1988). RSFA is sensitive to cerebrovascular and cardiovascular differences in ageing (Tsvetanov et al., 2021b), cerebrovascular disorders (Liu et al., 2021, Secchinato et al., 2019, Taneja et al., 2019), small vessel disease (Makedonov et al., 2013a), stroke (Nair et al., 2017), Alzheimer’s disease (Millar et al., 2020a), cognitive performance (Liu et al., 2022, Millar et al., 2020b; Millar et al., 2021; Millar et al., 2021) and the presence of brain tumours (Agarwal et al., 2019).

Combining acute and convalescent assessments with serology diagnosis, we report on the chronic effect of COVID-19 on RSFA as a marker of cerebral microvascular function, in relation to acute severity and in contrast to control group data. Acute disease severity was quantified by the COVID-19 WHO Progression Scale and blood biomarkers in patients hospitalised for COVID-19. We predicted that acute COVID-19 changes regional RSFA at follow up, in proportion to acute disease severity, over and above the effects of residual systemic cardiorespiratory impairment. A secondary hypothesis was that the level of RSFA abnormality would relate to worse functional, cognitive and mental dysfunction months after hospitalisation. We further examined the spatial correspondence between regional differences in RSFA and normative regional variations in neurotransmitters/receptors, brain energy consumption and cell-type distributions. We hypothesised that the physiological and genetic signature of COVID-19-related cerebrovascular impairment shapes the composition of cell-types, metabolism and vasoreactivity essential for neuronal homeostasis.

## 2 Methods

### 2.1 Participants

Patients were recruited through the NIHR COVID-19 BioResource, which received ethical approval from East of England – Cambridge Central Research Ethics Committee (REC 17/EE/0025), and provided written informed consent. Eligibility was based on admission to Addenbrooke’s Hospital, Cambridge UK with a serological diagnosis of COVID-19 between 10th March 2020 and 31st July 2020, aged 18 years or older, attended for outpatient visits following discharge, and no contraindications to MRI. 489 patients were potentially eligible. Clinical data were obtained from inpatient electronic medical records, and from cardiorespiratory and neurological assessments at follow-up clinical and research visits at least 6 weeks following symptom onset. 45 patients consented to participate and had clinical, structural magnetic resonance imaging (MRI) and resting state functional MRI (fMRI) data of appropriate quality (see below). Age and sex matched, non-hospitalised, non-COVID-19 controls (n = 42) were recruited by word of mouth and through the Cambridge NIHR BioResource (https://bioresource.nihr.ac.uk/centres-programmes/bioresource-centre-cambridge/). Controls were scanned with the same sequences, with data pooled over protocol-optimisation cohorts to match demographics (Cambridgeshire Research Ethics Committee 97/290 and REC 17/EE/0025, and Norfolk EE/0395 and a protocol approved by the Human Biology Ethics Committee of the Council of the School of Biological Sciences, University of Cambridge).

**Fig. 1:**
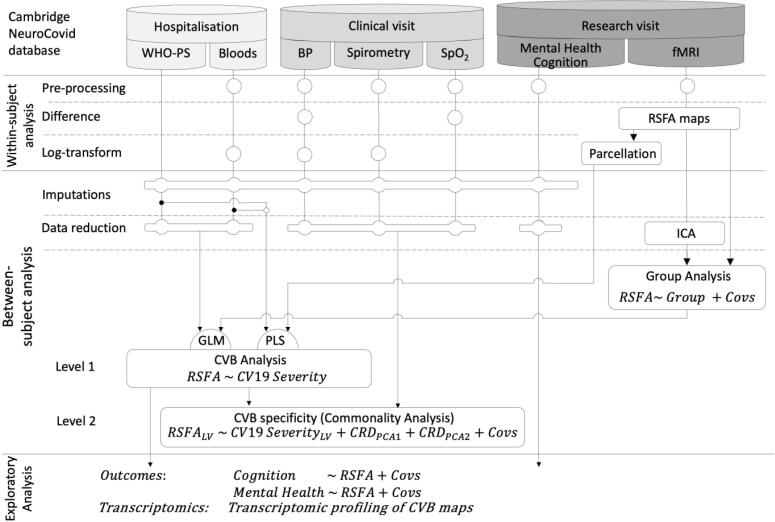
Schematic representation of various modality datasets in the study, their processing pipelines on a within-subject level, as well as data-reduction techniques and analytical strategy on between-subject level to test for associations between acute COVID-19 Severity and chronic cerebrovascular impairment. WHO-PS, COVID-19 WHO progression scale; BP, blood pressure; SpO2, blood oxygen saturation; fMRI, functional magnetic resonance imaging; RSFA, resting state fluctuation amplitudes; PCA, principal component analysis; ICA, independent component analysis; Covs, covariates of no interest; GLM, general linear model; PLS, partial least squares; LV, latent variable from PLS analysis; CRD, cardiorespiratory dysfunction component; CVB, cerebrovascular burden;

The study design and the principal data processing pipelines are summarised in Fig. 1.

### 2.2 Inpatient data: COVID-19 severity

The WHO COVID-19 11-point Progression scale was used during hospitalisation to provide a measure of disease severity with scores from 0 (non-infected) to 10 (dead) (Marshall et al., 2020). We also used blood biomarkers previously associated with COVID-19 severity including the most extreme values during hospitalisation on the following routine clinical blood tests: haematological cell counts (lowest platelets) (Wool and Miller, 2021); inflammatory and acute phase proteins (C-reactive protein, CRP, serum ferritin) (Luan et al., 2021) (interleukin-6, IL-6) (Group and Sterne, 2021, Ulhaq and Soraya, 2020); liver function tests (bilirubin) (Bangash et al., 2020); and blood coagulation markers (d-dimer, prothrombin time (PT) and activated partial thromboplastin time (APTT)) (Asakura and Ogawa, 2021, Iba et al., 2020, Levi et al., 2020). All blood-based measurements were positively skewed on natural scales and were log-transformed for closer approximation to Normal distributions before statistical analysis. For consistency in interpreting scores across blood assays, platelet counts were inverted (iPlatelets) so that higher scores represent lower counts. All variables were normalised to a mean of 0 and standard deviation of 1.

### 2.3 Clinical outpatient visit: Cardiorespiratory assessments

Cardiorespiratory measurements were collected during a clinical assessment at least 12 weeks after discharge from initial hospitalization with COVID-19 using Care Fusion Micro Spirometer (Care Fusion, San Diego, CA). Systolic and diastolic blood pressure (BPS and BPD) were measured in lying (or seated) and standing positions by automated sphygmomanometry. We calculated pulse pressure (BPS-BPD) and orthostatic intolerance (BPS lying – BPS standing). Lying and standing pulse pressure values were log-transformed for Normality prior to statistical analysis. Lung function test determined peak expiratory flow (PEF), forced expiratory volume in 1 s (FEV1), forced vital capacity (FVC) and FEV1/FVC ratio. Measurements were repeated in triplicate, with one minute rest between measurements and log-transformed for Normality prior to statistical analysis. Pulse oximetry was used to determine the heart rate and arterial oxygen saturation before and following a 6-minute walk test (du Bois et al., 2012, Crapo et al., 2012, Enright et al., 2003).

### 2.4 Research visit: neurological assessment

#### 2.4.1 Image acquisition and pre-processing

Imaging data were acquired using a 3T Siemens Prisma*fit* System with a 32–/20-channel head-coil at the Wolfson Brain Imaging Centre (WBIC; https://www.wbic.cam.ac.uk). A 3D-structural MRI was acquired on each participant using a T1-weighted sequence (3D Magenetisation-Prepared Rapid Gradient-Echo, 3D MPRAGE) with the following parameters: repetition time (TR) = 2 ms; echo time (TE) = 2.99 ms; inversion time (TI) = 880 ms; flip angle α = 9°; field of view (FOV) = 208×256×256 mm3; resolution = 1 mm isotropic; accelerated factor (in-plane acceleration iPAT) = 2; acquisition time, 5 min. T1 images were pre-processed using SPM12. The T1 image was rigid-body coregistered to the MNI template, and segmented to extract probabilistic maps of five tissue classes: gray matter (GM), white matter (WM), CSF, bone, soft tissue and residual noise.

RSFA was estimated from resting state Echo-Planar Imaging (*EPI*) of 477 volumes acquired with 64 slices for whole brain coverage (TR = 735 ms; TE = 30 ms; FOV = 210 mm × 210mm; resolution = 2.38 × 2.38x2.4 mm) during 5 min and 51 s. Participants were instructed to lie still, to stay awake and keep their eyes open, looking at a fixation cross. *EPI* data preprocessing included the following steps: (1) temporal realignment of slices to (0,0,0) Montreal Neurological Institute (MNI) co-ordinates; (2) spatial realignment to adjust for linear head motion; (3) identification and censoring (scrubbing) of outlier scans using Artifact detection tools (ART, https://www.nitrc.org/projects/artifact_detect/); (4) rigid-body coregistration to the T1 anatomical image; and (5) application of the normalization parameters derived from T1 image coregistration to warp the functional images into MNI space. We applied whole-brain independent component analysis for single subject time series denoising to minimise motion artefacts using *a priori* heuristics implemented in the ICA-based Automatic Removal of Motion Artifact toolbox (Pruim et al., 2015b, Pruim et al., 2015a) after smoothing with a 6 mm FWHM Gaussian kernel.

RSFA maps were estimates using previously reported procedure (Tsvetanov et al., 2021b, Tsvetanov et al., 2015).To facilitate integrative multivariate analyses (see below), the RSFA maps were parcellated by a prior cortical template into 360 bilaterally symmetric regions (Glasser et al., 2016). Regional RSFA values were estimated by averaging over all voxels in each parcel.

#### 2.4.2 Physical, cognitive and mental dysfunction (PMC)

Quality of life, cognition and mental health were assessed using a set of questionnaires: Generalised Anxiety Disorder-7 (GAD-7) (Spitzer et al., 2006, Swinson, 2006), Patient Health Questionnaire-9 (PHQ-9) (Kroenke et al., 2001), Patient Health Questionnaire-15 (PHQ-15) (Kroenke et al., 2002), Posttraumatic Stress Disorder Checklist-5 (PCL-5) (Blevins et al., 2015) and subscores from the Short Form-36 (SF-36) (Ware and Sherbourne, 1992). SF36 subscores were defined as physical functioning (SF36-PF), role limitation physical (SF36-RLP), role limitation emotional (SF36-RLE), energy dimension (SF36-ED), emotional wellbeing (SF36-EW), social functioning (SF36-SF), pain (SF36-P) and general health (SF36-GH).

Cognitive function and functional independence were evaluated using Montreal Cognitive Assessment (MOCA) (Nasreddine et al., 2005), inverted Modified Ranking Scale (iMRS) (Eriksson et al., 2007) and Barthel Index (BI) (Mahoney and Barthel, 1965). For consistency in interpreting scores across questionnaires, scores for mental health questionnaires were inverted (iGAD-7, iPHQ-9, iPHQ-15 and iPCL-5) so that lower values represent greater mental health problems.

### 2.5 Analytical approach

#### 2.5.1 Group differences in cerebral microvascular health

To study differences in cerebral microvascular health between COVID-19 cases and controls, we performed independent component analysis on the imaging-based RSFA data to separate spatially overlapping sources of signal with different aetiologies (Xu et al., 2013), as it is known that cardiovascular versus cerebrovascular signals may vary across individuals and brain region in RSFA (Tsvetanov et al., 2021b). The independent component analysis was performed across participants to determine spatially non-overlapping RSFA maps without using group information, termed Source-Based Cerebrovasculometry (Tsvetanov et al., 2021b, Tsvetanov et al., 2015) with ICASSO (software for investigating the reliability of ICA estimates by clustering and visualisation; Himberg and Hyvarinen, 2003) across 128 iterations using the Group ICA of fMRI Toolbox (https://mialab.mrn.org/software/gift/index.html; Calhoun et al., 2001). In brief, the fastICA algorithm was applied after the optimal number of sources explaining the variance in the data was identified using PCA with Minimum Description Length (MDL) criterion (Hui et al., 2011, Li et al., 2007, Rissanen, 1978). By combining the PCA and ICA, the concatenated RSFA maps in a *n*-by-m matrix of participants-to-voxels are decomposed into: (i) a set of maximally independent components, each characterized by a different, cerebrovasculometry source map, showing the spatial projection of the component to each brain voxel (termed RSFA_IC_ maps), and (ii) the degree to which each participant expresses the spatial map of the corresponding component (RSFA_IC_ map), termed subject scores. Subject scores for each component were predicted by diagnostic status (COVID-19 cases vs controls), age, and sex in a subsequent robust multiple linear regression. The regression model was specified by Wilkinson’s notation, *‘RSFA*_*IC*_ ∼ 1 + group*age + sex’ and fitted for each component separately. Models were corrected for multiple comparisons at p < 0.05 (FDR-corrected). To confirm the validity of the results a univariate voxel-wise analyses was performed.

#### 2.5.2 Linking COVID-19 severity to cerebrovascular impairment and their correlates

Rate of missing inpatient, outpatient and research visit data varied between 0 and 38 % (see Table 1); hence, to increase statistical power and efficiency, missing data were imputed before further statistical analyses (see below). Incomplete variables were imputed under fully conditional specification, using the default settings of the multivariate imputation by chained equations (MICE) in R (van Buuren and Groothuis-Oudshoorn, 2011). Multiple versions of each dataset were created (m = 5). Instead of accounting for variability in the parameter estimates between imputed datasets, we report any differences in the significance of parameters input to the multiple linear regression.

**Table 1:** Characteristics of 45 cases hospitalised with COVID-19. n – indicates number of patients and the percentage of patients from the patient cohort (%), SD – standard deviation, IQR – interquartile range, kg – weight in kilograms, m – height in meters.

Variable		Patients	
		n (%)	Mean(SD)/Median(IQR)*
Age (years)		45 (100)	52 (14)
Education (years)		45 (100)	16 (4)
Female		26 (58)	–
Right-handed		40 (89)	–

Pre-existing comorbidies (n > 1)			
	Cardiovascular	11 (24)	–
	Respiraroty	8 (18)	–
	Type-2 Diabetes	4 (9)	–
	Hypothyroidism	4 (9)	–
	Neurological	2 (4)	–

Inpatient data			
	Vasopressors	10 (22)	–
	Dialysis	4 (9)	–
	BMI (kg/m2)	36 (80)	29 (4)
	Mechanical Ventilation (days)	13 (29)	20 (15)
	CRP	42 (93)	149 (147)
	D Dimer	37 (82)	1125 (2071)
	Ferritin	32 (71)	1225 (1700)
	IL-6	28 (62)	39 (80)
	PT	32 (71)	26 (54)
	APTT	33 (73)	42 (36)
	Bilirubin	43 (96)	14 (8)
	Platelets	44 (98)	230 (102)

Clinical Visit			
	HR (bpm)	38 (84)	76 (14)
	RR (cpm)	38 (84)	16 (2)
	SBP lying (mmHg)	38 (84)	134 (23)
	SBP standing (mmHg)	33 (73)	130 (20)
	DBP lying (mmHg)	38 (84)	74 (12)
	DBP standing (mmHg)	33 (73)	79 (11)

Research Visit			
	GAD-7 (0–21)	44 (98)	4 (8)*
	PHQ-9 (0–27)	44 (98)	7 (9)*
	PHHQ-15 (0–30)	39 (87)	9 (8)*
	PCL-5 (0–80)	44 (98)	16 (22)*
	MoCA	39 (87)	28 (3)*
	mRS	39 (87)	1 (1)*
	BI	37 (82)	20 (0)*

Initial symptoms to Admission (days)		40 (89)	14 (14)
Hospitalisation Duration (days)		40 (89)	19 (31)
Intial symptoms to Clinic visit (days)		39 (87)	169 (35)
Intial symptoms to Research visit (days)		45 (100)	180 (58)

Statistical analyses used Matlab 2020b calling the packages as described below. Datasets of interest stemmed from a range of modalities and different time-points: i) inpatient blood samples, ii) outpatient cardiorespiratory dysfunction, iii) mental health and RSFA measures during a research visit. To make these datasets tractable for univariate stages of the two-level analytical procedure (Passamonti et al., 2019, Tsvetanov et al., 2021a, Tsvetanov et al., 2018, Tsvetanov et al., 2016). In the first-level analysis, the link between COVID-19 severity and RSFA data was identified using a multivariate approach (see below). In the second-level analysis, we tested whether the COVID-19-RSFA link can be explained by other variables of interest or potential covariates of no interest using regression-based analyses (see below). We therefore constructed a set of summary measures for each non-imaging modality (i.e. components, Fig. 1). This had two advantages. First, it reduced the number of statistical comparisons. Second, it improved interpretability of the signals in each modality data by means of denoising and simplification given; low-dimensional data representations remove noise but retain signal of interest that can be instrumental in understanding hidden structures and patterns of multivariate data. We performed principal component analysis as a widely used dimensionality reducing approach (Hotelling, 1933, Pearson, 1901, van der Maaten et al., 2009) on each set of non-imaging variables from clinical and research assessments data before integrating them with MRI data as described below. For this purpose, we used Matlab’s function *pca.m* with default settings, where the number of components was determined using Horn’s parallel analysis (Dobriban, 2020, Horn, 1965) using 10,000 permutations. All variables were normalised to a mean of 0 and standard deviation of 1 prior principal component analysis.

##### 2.5.2.1 Chronic cardiorespiratory dysfunction

Cardiorespiratory dysfunction was represented by the first two principal components (CRD_PC1_ and CRD_PC2_) constructed from blood pressure (lying and standing pulse pressure, and orthostatic systolic and diastolic blood pressure), differences in heart rate and blood oxygenation during a 6-minute walk test, and a spirometry pulmonary function component.

##### 2.5.2.2 Chronic physical, cognitive, and mental dysfunction (PCM)

Chronic physical, cognitive and mental dysfunction (PCM) was represented by the first two principal components (PCM_PC1_ and PCM_PC2_) constructed from mental health scores (GAD-7, PHQ-9, PHQ-15, and SF-36 sub-scores), cognitive function (MoCA) and functional independence measures (Barthel Index and imRS).

##### 2.5.2.3 Two-level approach linking COVID-19 severity to cerebrovascular impairment and their correlates

The prediction of RSFA abnormality by prior clinical assessment of COVID-19 severity was tested using a multivariate approach using a two-level procedure (Passamonti et al., 2019, Tsvetanov et al., 2021a, Tsvetanov et al., 2018, Tsvetanov et al., 2016). First, the relationships between COVID-19 severity and RSFA data were identified using partial least squares (Krishnan et al., 2011) of RSFA maps and COVID-19 Severity data, by providing pairs of latent variables (RSFA_LV_) and (COVID-19 Severity_LV_). Data set 1 consisted of parcellated RSFA maps across all patients (45 cases × 360 brain regions; RSFA dataset). Data set 2 included the COVID-19 WHO Progression scale and inpatient blood assay results (45 cases × 9 clinical severity measures; COVID-19 Severity dataset). All variables were Z-scored (mean of 0 and standard deviation of 1) before PLS with 10,000 random permutations of dataset 2 to determine the significance of the latent variables. To confirm the validity of the results, a univariate voxel-wise analyses was performed.

Second, we tested whether the identified relationship between COVID-19 Severity_LV_ and RSFA_LV_ could be explained by other variables of interest or potential confounding variables of no interest useing robust multiple linear regression and commonality analysis (Kraha et al., 2012, Nimon et al., 2008). Commonality analysis partitions the variance explained by all predictors in a multiple linear regression model into variance unique to each predictor and variance shared between each combination of predictors. Therefore, unique effects indicate the (orthogonal) variance explained by one predictor over and above that explained by other predictors in the model, while common effects indicate the variance shared between correlated predictors. Notably, the sum of variances, also known as commonality coefficients, equals the total proportion of variance explained R2 by the regression model. We adapted a commonality analysis algorithm (Nimon et al., 2008) implemented in Matlab (Wu et al., 2023). This model tested whether the relationship between COVID-19 Severity_LV_ and RSFA_LV_ can be explained partly or fully by systemic cardiorespiratory dysfunction or other covariates of no interest. The regression model was specified by Wilkinson’s notation *RSFA_LV_ ∼ 1 + COVID-19 Severity_LV_ + CRD_PC1_ + CRD_PC2_ + Age + Sex*. The model can therefore identify unique variance explained by each of the predictors, i.e.,whether COVID-19 Severity_LV_ predicts RSFA_LV_ over and above other predictors. Common effects of interest were the cardiorespiratory-related effects, defined by the common variance between COVID-19 Severity_LV_ and CRD_PC1_ and CRD_PC2_. Significant effects were identified by nonparametric testing using 10,000 permutations using commonality analysis implementation in Matlab (Wu et al., 2023).

##### 2.5.2.4 COVID-19-related cerebrovascular impairment associations with physical, cognitive, and mental dysfunction

COVID-19-related abnormalities in RSFA identified in the first-level analysis, were related to the two principal components of physical, cognitive, and mental functioning (PCM_PC1_ and PCM_PC2_) using robust regression. The PCM components were defined as dependent variables in separate models. RSFA_LV_, age and sex were entered as predictors. The model formulas were specified by Wilkinson’s notation, ‘*PCM ∼ 1 + RSFA*_*LV*_ *+ age + sex*’ and fitted for the two PCM components separately. To confirm the validity of the results for the significant models, a univariate voxel-wise analyses was performed.

##### 2.5.2.5 Spatial covariance of COVID-19-related cerebrovascular impairment with regional neurotransmitter, metabolic and cell-type distribution

We further assessed the spatial overlap between COVID-19-related cerebrovascular burden map and a range of brain metabolic, neurotransmitter, gene expression and cell-type parameters, including i) existing receptor/metabolic templates and ii) gene transcription profiling maps. Templates of interest included metabolic rates of glucose, oxygen, and aerobic glycolysis (Vaishnavi et al., 2010) and receptor and transmitter maps across nine different neurotransmitter systems (Hansen et al., 2021b), all measured by positron emission tomography (PET). Gene expression maps (Hawrylycz et al., 2012) were based on key proteins implicated in SARS-Cov-2 cellular attachment (angiotensin converting enzyme-2, ACE2; neuropilin-1, NRP1; neuropilin-2, NRP2), proteolytic processing (cathepsin-B, CTSB; cathepsin-L, CTSL) and viral defence (interferon type 2 receptors, IFNAR2; lymphocyte antigen 6-family member E, LY6E) (Iadecola et al., 2020, Yang et al., 2021). Spatial correlations were evaluated using 10,000 spin-based permutation tests (p-spin) preserving spatial autocorrelation (Alexander-Bloch et al., 2018, Fulcher et al., 2021).

Spatial covariance between the cerebrovascular burden map and gene expression across 9394 genes expressed in the human brain (Hawrylycz et al., 2012) was based on PLS association using spin-premutation-based 10-fold cross-validations (Tsvetanov et al., 2021a). The latent variable represented a spatial pattern of gene expression that covaried significantly with the cerebrovascular burden pattern associated with COVID-19 severity. Full details about the processed transcriptomic data are available elsewhere (Arnatkevic̆iūtė et al., 2019). Genes highly expressing this pattern (i.e. high loadings) were tested against a molecular atlas of human brain vasculature of 17 control and Alzheimer’s disease patients (Yang et al., 2021) using cell-type decomposition (Hansen et al., 2021). This enabled us to test whether the cerebrovascular burden-relevant genes were preferentially expressed in specific cell types, i.e., testing for gene sets specific to eleven major canonical cortical cell classes: astrocytes (Astro); brain endothelial cells (BEC); ependymal cells (Epend); macrophage/microglia (MacMic); meningeal fibroblasts (MFibro); neurons (Neuro); oligodendrocyte precursors (Opc); oligodendrocytes (Oligo); pericytes (*Peri*); perivascular fibroblasts (PFibro); and smooth muscle cells (SMC). To this end, we calculated the ratio of genes in each set preferentially expressed by each cell type (e.g. ratio for pericytes is calculated from the number of genes preferentially expressed in pericytes divided by the total number of genes). Gene sets were thresholded to include the top *n*% of genes with greatest loadings, where *n* varied from 10 % to 100 % (all genes). Statistical significance was determined using a null distribution of ratios based on 10,000 sets of random genes (Hansen et al., 2021).

### 2.6 Data availability and code

Code and composite data to reproduce manuscript figures and statistical analyses are available at https://github.com/kamentsvetanov/covid19_cerebrovascularburden. Resting-state fMRI data were pre-processed using SPM12 and post-processed using a GLM-like approach (Geerligs et al., 2017) available at https://github.com/MRC-CBU/riksneurotools/blob/master/GLM/. MATLAB-based commonality analysis for neuroimaging (Wu et al., 2021) is available at https://github.com/kamentsvetanov/CommonalityAnalysis/. Visualisation of neuroimaging results was in MRIcroGL and BrainSpace. Neurotransmitter receptor and transporter maps were available at https://github.com/netneurolab/hansen_receptors. Spin permutations used code available at https://github.com/frantisekvasa/rotate_parcellation. Fully-pre-processed transcriptomic data were available at https://figshare.com/articles/dataset/AHBAdata/6852911 and https://github.com/BMHLab/AHBAprocessing. The molecular atlas of the human brain vasculature was available at https://www.biorxiv.org/content/https://doi.org/10.1101/2021.04.26.441262v1. Code for cell-type decomposition analysis was available at https://github.com/netneurolab/hansen_genescognition.

## 3 Results

### 3.1 Participants

Characteristics of the 45 cases hospitalized with COVID-19 are detailed in Table 1. At hospitalisation, 30 % of the patients required mechanical ventilation (n = 13) for an average of 20 days; 22 % of patients required vasopressors (n = 10), and 9 % required renal replacement therapy with continuous veno-venous haemodiafiltration (n = 4). Most common pre-existing comorbidities at admission with Covid-19 were cardiovascular dysfunction (n = 11, 24.4 %; hypertension, n = 10; Von Willbrand disease type 1, n = 1), respiratory disorders (n = 8, 17.8 %), type-2 diabetes (n = 4, 8.9 %), hypothyroidism (n = 4, 8.9 %), migraine (n = 2, 4,4%). Other single case conditions included obesity, type-1 diabetes, rheumatoid arthritis, dyslipidaemia, De Quervain’s thyroiditis, ulcerative colitis, prostatic arthritis, acute lymphoblastic leukaemia, osteoarthritis, hysterectomy, appendicectomy, acute kidney disease, and non-alcoholic fatty liver disease. The average number of days from initial symptoms to clinical out-patient and research assessments was 169 ± 35 and 180 ± 58, respectively. The percentage of missing values across the inpatient, clinical visit and research visit variables varied between 0 and 38 %. In total 69 out of 405 records (17 %) were incomplete for impatient data; 71 out of 360 records (20 %) were incomplete for clinical visit data; and 121 out of 675 records (18 %) were incomplete for cognitive and mental health data.

### 3.2 Group differences in cerebrovascular components

**Fig. 2:**
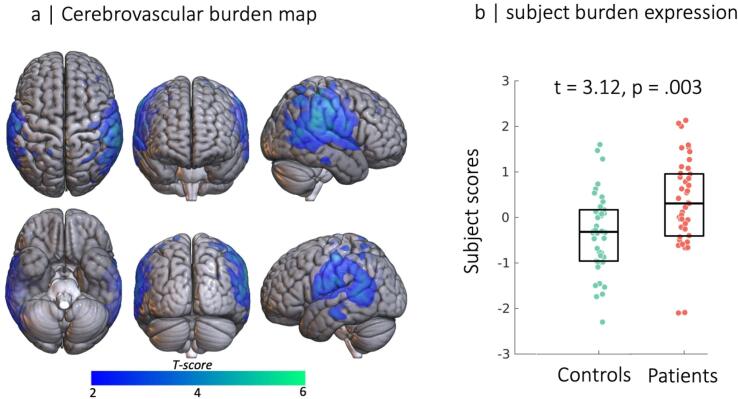
Group differences in RSFA. Source-based cerebrovasculometry for the component differentially expressed between groups: (**a**) independent component spatial map reflecting decrease in RSFA values in temporo-parietal regions. (**b**) Box plots of subject scores for patients hospitalised for COVID-19 (red) and control group (green, each circle represents an individual) indicating higher loading values for patients than controls as informed by two-sample unpaired permutation test (a robust regression was used to down-weight the effects of extreme data points). (For interpretation of the references to colour in this figure legend, the reader is referred to the web version of this article.)

The decomposition of RSFA with source-based cerebrovasculometry resulted in 10 spatially independent components according to the MDL criterion with near perfect stability indices across 128 ICASSO iterations (mean of 0.97 and standard deviation of 0.01). One component showed significant difference between the patient and control groups in terms of their subject scores (RSFA_IC4_, t = 3.12, p = 0.003, Fig. 2), while controlling for age and sex in a robust linear regression. The spatial map of this component, labelled RSFA_IC4_, included voxels with high values in temporo-parietal regions, indicating that individuals with higher loading values, in this case the patient group, had lower RSFA values in these regions, relative to the control group (Fig. 2). The other components did not differentiate patients from controls.

**Fig. 3:**
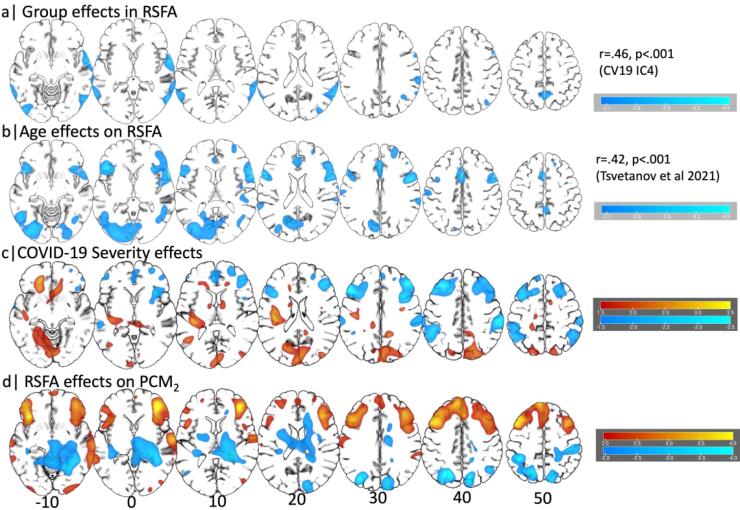
Voxel-wise association in RSFA data. Association between RSFA and group identity (patient vs control) **(a)** To confirm the validity of results from the source-based cerebrovasculometry, we performed a second-level univariate analysis in SPM12 with RSFA as dependent variable. Group identity, age and sex were defined as predictors. The spatial pattern of group effects was highly consistent with the pattern identified using ICA (IC4), r = 0.46, p-spin < 0.001. **(b)** The spatial pattern associated with age effect was highly consistent with the pattern derived from a previous study using a large population-based cohort (n = 226, Tsvetanov et al., 2021b), r = 0.42, p-spin < 0.001. **(c)** Association between COVID-19 Severity PC1 (see Fig. 5A) and RSFA on voxel level showing a negative association between COVID-19 Severity and RSFA values in frontal and temporoparietal regions confirming the validity of the results in the partial-least squares analysis capturing the multivariate relationship between COVID-19 severity and cerebrovascular impairment. This was based on a second-level univariate analysis in SPM12 with RSFA as dependent variable. COVID-19 severity component, age and sex were defined as predictors. The nine measures constructing the COVID-19 severity component (COVID-19 Severity_PC1_) included COVID-19 WHO Progression Scale and blood markers (CRP, ferritin, IL-6, bilirubin, d-dimer, PT, APTT and iPlatelets), see Fig. 5A. The model can be represented using Wilkinson’s notation as follows: *RSFA ∼ 1 + COVID-19 Severity*_*PC1*_*+ Age + Sex*. **(d)** Association between the second physical, cognitive, and mental functioning component (PCM_PC2_) and RSFA on voxel level showing a positive association between phsycial and cognitive functioning with RSFA values in frontal and temporall regions. Maps are thresholded at uncorrected p-values of 0.05 for more complete description of the spatial representation.

The spatial pattern of RSFA_IC4_ was consistent with the univariate voxel-wise approach (r = 0.46, p < 0.001, Fig. 3A). In addition, the univariate approach revealed that the spatial pattern in RSFA associated with age was highly consistent with the one reported on large-scale population-based cohorts, r = 0.42, p < 0.001 (Tsvetanov et al., 2021b, Tsvetanov et al., 2015). This suggests that RSFA can detect reliably differences in cerebrovascular health across various phenotypes in smaller samples. Though age is a risk factor for COVID-19 severity (Verity et al., 2020) and RSFA (Tsvetanov et al., 2021b, Tsvetanov et al., 2015), the COVID-19 group effect was not explained by individual’s age, and showed only a partial overlap with the effects of age on RSFA in parietal regions (Fig. 3B) (Tsvetanov et al., 2021b, Tsvetanov et al., 2015).

### 3.3 COVID-19 severity predicts cerebrovascular impairment

**Fig. 4:**
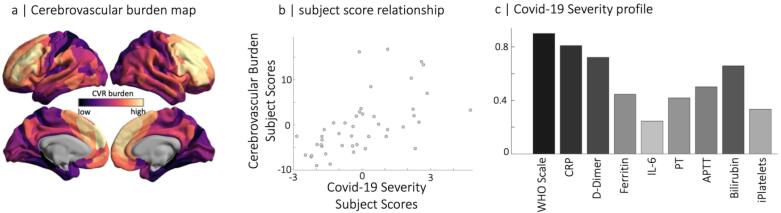
Link between COVID-19 severity and RSFA. Partial least squares analysis of COVID-19 severity data at acute stage and RSFA-based cerebrovascular burden (CVB) at chronic stage. (**a**) Spatial distribution of parcellated RSFA values where dark to light colours are used for the strength of positive and negative correlations with the COVID-19 Severity profile (**c**). Note that regions with high cerebrovascular burden have low values in RSFA. (**b**) The scatter plot in the middle panel represents the relationship between subjects scores of RSFA-latent variable and COVID-19 Severity-latent variable identified by partial least squares analysis.

Using PLS analysis, we identified one significant pair of latent variables (r = 0.595, p = 0.011, based on a null distribution of 10,000 permutations). Variable loadings and subject scores reflecting the strong relationship between acute COVID-19 Severity and chronic RSFA abnormalities are shown in Fig. 4.

**Fig. 5:**
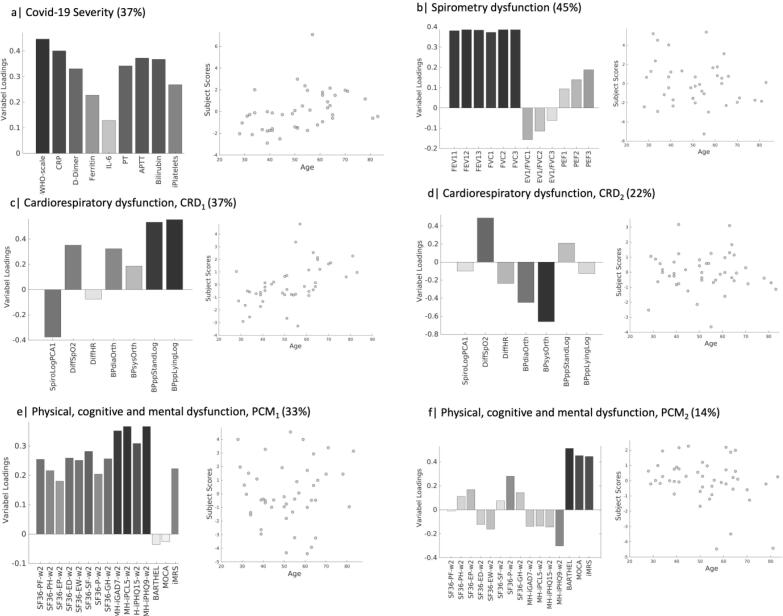
Data reduction of inpatient, clinical and research visit non-neuroimaging data using principal component analysis (separately on each dataset). **(a)** COVID-19 Severity component explaining 40% of the total variance loading most strongly on WHO COVID-19 11-point Progression scale (WHO-scale), C-reactive protein (CRP), d-dimer, ferritin, followed by bilirubin and activated partial thromboplastin time (APTT). Other biomarkers [interleukin-6, prothrombin time (PT), platelets (iPlatelets, counts so that higher scores represent lower counts)] loaded in the expected direction, but to a lesser extent. Scatter plots of subject scores for the corresponding components versus chronological age, where each circle is one patient. **(b)** Reduction of spirometry measures to a single variable to balance the representativeness of each data type for cardiorespiratory dysfunction dimensionality reduction (Hastie et al., 2009). Of the 12 spirometry measures the first principal component expressed FEV1 and FVC values explaining 45% of the spirometry data. FEV1 – forced expiratory volume in 1 s; PEF – peak expiratory flow; FVC – forced vital capacity. **(c)** First cardiorespiratory dysfunction component (CRD_1_) constructed from chronic cardiorespiratory data, explaining 37% and loading highly on lung function, oxygen saturation and pulse pressure. **(d)** The second component, explaining 22%, loaded on oxygen saturation and orthostatic hypotension. SpiroLogPCA1 – first principal component across 12 log-transformed spirometry variables (see panel **b**); DiffSpO2 and DiffHR – difference in arterial oxygen saturation and heart rate before and after a 6-minute walk test; BPdiaOrth and BPsysOrth – orthostatic intolerance in diastolic and systolic blood pressure, respectively; BPppStandLog and BPppLyingLog – pulse pressure while standing and lying, respectively. **(e)** The first component of physical, cognitive and mental dysfunction (PCM_1_) explaining 33% and loading highly on mental health variables. **(f)** PCM_2_ explaining 14% and loading positively on cognitive function and functional independence. SF36-PF – physical functioning; SF36-RLP – role limitation physical, SF36-RLE – role limitation emotional, SF36-ED – energy dimension, SF36-EW – emotional wellbeing, SF36-SF – social functioning, SF-P – pain; SF36-GH – general health; iGAD7 – Generalised Anxiety Disorder-7; PCL5 – Posttraumatic Stress Disorder Checklist-5; PHQ15 – Patient Health Questionnaire-15; PHQ9 – Patient Health Questionnaire-9; BARTHEL – Barthel Index; MOCA – Montreal Cognitive Assesment; iMRS – inverted Modified Ranking Scale;

The RSFA latent variable (RSFA_LV_) expressed negative loadings in frontal (superior frontal gyrus, middle frontal gyrus, inferior frontal gyrus and portions of the anterior cingulate) and parieto-temporal (angular gyrus, supramarginal gyrus, superior temporal gurus, middle temporal gyrus) regions. This pattern of COVID-19 Severity-related reduction in RSFA values was mirrored in a voxel-wise analysis of RSFA maps and the only significant COVID-19 Severity component (p < 0.001) in the inpatient data (Fig. 3C and Fig. 5A, respectively). Positive loadings in the RSFA data appeared to be in postcentral gyrus, calcarine sulcus, cuneus, and lingual gyrus. Increase in the RSFA signal in these regions may reflect increased pulsatility in neighbouring vascular and white matter territories as reported previously (Makedonov et al., 2016, Makedonov et al., 2013b, Tsvetanov et al., 2021b, Tsvetanov et al., 2015). This pair of latent variables suggested that patients with higher COVID-19 Severity at the acute stage have sustained changes in cerebrovascular function in frontal and temporo-parietal regions after discharge from hospital. For visualisation purposes we inverted the loading values in Fig. 4A so that higher values reflect poorer cerebrovascular function, i.e. higher cerebrovascular burden.

To understand whether the relationship between regional RSFA impairment and COVID-19 Severity can be explained by the components of chronic systemic cardiorespiratory dysfunction or covariates of no interest, we performed a second level robust regression analysis. Cardiorespiratory dysfunction was represented by two significant components (p < 0.001 and p < 0.001; Fig. 5C and D). The first component, CRD_PC1_, represented overall cardiorespiratory dysfunction in terms of poor lung function, poor oxygen saturation coupled with high pulse pressure. The second component, CRD_PC2_, represented a more specific component of cardiorespiratory dysfunction in terms of oxygen desaturation and orthostatic hypotension. CRD_PC2_ likely reflects patient differences in sympathetic failure, which is associated with orthostatic fall in blood pressure and oxygenation (Jakobsen et al., 2017). Overall, the two CRD components are consistent with the idea of cardiovascular health being multifactorial, where autonomic nervous system response is clearly dissociated from the two components of blood pressure (King et al., 2022, Tsvetanov et al., 2021b). The regression model specification was based on the following syntax using Wilkinson notation: *RSFA_LV_* ∼ 1 + *COVID-19Severity_LV_ + CRD_PCA1_ + CRD_PCA2_ + Age + Sex*. COVID-19 Severity was the only significant predictor of RSFA*_LV_* in the model (r = 0.490, p < 0.001), suggesting that chronic cardiorespiratory dysfunction, age and sex cannot explain fully the relationship between COVID-19 Severity and RSFA abnormality. None of the pre-existing comorbidity conditions explained this relationship (e.g. by adding comorbidity categories as covariates of no interest, COVID-19 Severity remained highliy significant predictor, r = 0.451, p < 0.001; noting that having a respiratory condition was independently associated with RSFA_LV_, r = 0.336, p = 0.004). Interestingly, the unique variance explained by COVID-19 Severity in the regression model was weaker than the variance identified by the PLS analysis (r = 0.595 vs r = 0.490 for PLS and MLR analyses) suggesting that one or more of the predictors in the model explain some of the covariance between COVID-19 Severity and RSFA. Permutation-based commonality analysis with 10,000 permutations confirmed that a portion of the variance between COVID-19 Severity_LV_ and RSFA_LV_ was explained uniquely by age (13 % total, p < 0.001) or cardiorespiratory dysfunction component 2 (CRD_PC2_, 4 % total, p = 0.002), or by shared effects of age and cardiorespiratory dysfunction component 1 (Age, CRD_PC1_, 18 % total, p < 0.001), SI Table 1. COVID-19 Severity_LV_ remained as the largest unique predictor of variance in RSFA_LV_ (40 % total, p = 0.004).

### 3.4 COVID-19-related cerebrovascular burden association with physical, cognitive, and mental functioning

The level of RSFA abnormalities (RSFA_LV_) was related to physical, cognitive and mental dysfunction represented by two significant components (p < 0.001 and p < 0.001). The first component, PCM_PC1_, expressed highly mental health variables (GAD-7, PCL-5, PHQ-9, PHQ-15 and SF-36 sub-scores), thus reflecting overall mental health (PCM_PC1_, Fig. 5E). The second component, PCM_PC2_, expressed highly cognitive function and functional independence variables (Barthel Index, MoCA and iMRS, Fig. 5F). RSFA_LV_, age and sex were entered as predictors, while PCM components were used as dependent variables in separate robust regression model (Model 1: *PCM_PCA1_ ∼ 1 + RSFA_LV_ + age + sex*; Model 2: *PCM*_*PCA2*_ *∼ 1 + RSFA*_*LV*_ *+ age + sex*). Model 1 was not significant (p = 0.447), while Model 2 was significant (R2 = 0.303, p = 0.002) with RSFA significantly related to PCM_PC2_ (r = -0.362, p = 0.010). This indicates that patients with higher RSFA abnormality have worse cognitive function and less functional independence. None of the pre-existing comorbidity conditions explained this relationship (e.g. by adding comorbidity categories as covariates of no interest), RSFA remained significant predictor of PCM_PC2_, r = -0.550, p = 0.001. The findings in Model 2 were confirmed using voxel-wise analysis on RSFA maps, instead of RSFA_LV_ (Fig. 3D).

### 3.5 Spatial overlap of COVID-19-related cerebrovascular impairment with brain neurotransmitter and metabolic distribution

**Fig. 6:**
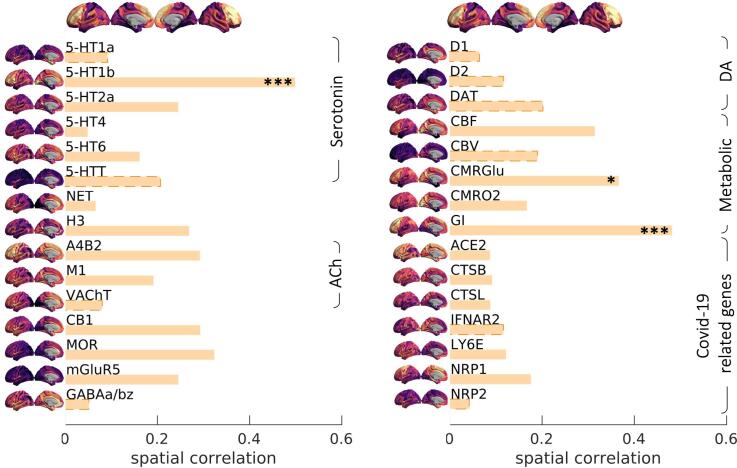
Spatial correspondence between COVID-19-related cerebrovascular burden map with neurotransmitter and brain distributions. Spatial correlation between Covid19 severity-induced cerebrovascular burden map and spatial patterns associated with a range of neurotransmitter receptor/transporters (Hansen et al., 2021b), selected genes relevant to SARS-CoV-2 brain entry (Iadecola et al., 2020) and brain metabolism parameters (Vaishnavi et al., 2010). Neurotransmitter receptors and transporters were selective to serotonin (5-HT1a, 5-HT1b, 5-HT2a, 5-HT4, 5-HT6, 5-HTT), norepinephrine (NET), histamine (H3), acetylcholine (ACh, A4B2, M1, VAChT), cannabinoid (CB1), opioid (MOR), glutamate (mGluR5), GABA (GABAa/bz) and dopamine (D1, D2, DAT). Metabolic maps were based on cerebal blood flow (CBF), cerebral blood volume (CBV), cerebral metabolic rate of glucose and oxygen (CMRGlu, CMRO2) and glycemic index (GI). Selective genes relevant to SARS-CoV-2 brain entry included angiotensin converting enzyme-2, ACE2; neuropilin-1, NRP1; neuropilin-2, NRP2, cathepsin-B, CTSB; cathepsin-L, CTSL, interferon type 2 receptors, IFNAR2; lymphocyte antigen 6-family member E, LY6E. The spatial maps of 5-HT1b, CMRGlu and Glycemic Index (GI) were significantly correlated with Covid19 severity-induced cerebrovascular burden map (* p-spin < 0.05 (one-sided), *** p-spin < 0.001). See text for more information.

We next assessed the spatial overlap between COVID-19-related cerebrovascular burden maps with existing neurotransmitter and metabolic maps using spatial autocorrelation-preserving permutation testing (Alexander-Bloch et al., 2018). Across 21 candidate maps, we show that the cerebrovascular burden map overlaps with the distribution of serotonin’s vasoactive receptor 5-HT1b (r = 0.52, p-spin < 0.001), aerobic glycolysis (r = 0.51, p-spin < 0.001) and to a weaker extent cerebral metabolic rate of glucose in the brain (r = 0.38, p-spin_one-sided_ = 0.041). However, the regional distribution of RSFA abnormality showed little correlation with the expression of key proteins implicated in SARS-CoV-2 cellular attachment, processing and viral defence (Fig. 6). Collectively, these results demonstrate that the distribution of cerebrovascular impairment related to COVID-19 severity is aligned with the spatial distribution of receptors and processes involved in the coordination of metabolic and vasoreactive responses.

**Fig. 7:**
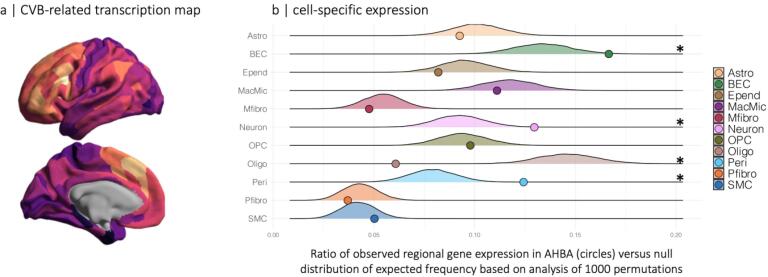
Spatial correspondence between COVID-19-related cerebrovascular burden and cell-type decomposition. (**a**) Spatial map of the weighted whole genome expression profile correlated with the COVID-19-induced cerebrovascular burden map (CVB). (**b**) Cell-type decomposition was used to identify cell-type enrichment based on extent to which genes expressed the transcriptome map in a. Gene sets for each cell-type was constructed by thresholding the top 70 % of genes with greatest loadings. Note that results were consistent across a range of thresholds, ranging from 10 % to no threshold. The ratio of genes in each gene set preferentially expressed in eleven distinct cell-types (circles) is shown against their null distribution of a model with random selection of all genes (10,000 permutations, *p-value < 0.05). For example, pericyte’s ratio is calculated from the number of genes preferentially expressed in pericytes divided by the total number of genes. Cell type-specificity of genes is described elsewhere (Yang et al., 2021) Astro – astrocytes, BEC – brain endothelial cells, Epend – ependymal, MacMic – macrophage/microglia, Mfibro – meningeal fibroblast, Neuron – neuron, OPC – oligodendrocyte precursor cells, Oligo – oligodendrocytes, *Peri* – pericytes, Pfibro – perivascular fibroblast, SMC – smooth muscle cells.

As a final step, we used normative transcriptomics to identify genes that are normally preferentially expressed in the regions associated with COVID-19-induced cerebrovascular impairment. Regularised-PLS identified one latent component (r = 0.64, p = 0.001, Fig. 7A). Using cell-type decomposition analysis on vascular cell-type specific gene sets, we determined the ratio of genes in each gene set preferentially expressed across eleven cortical cell types. Gene-sets were thresholded to include the top 70 % of genes with the highest loadings (Fig. 7B). While the 70 % threshold was arbitrary, that results were consistent across thresholds ranging from 10 to no threshold. Highly ranking genes were significantly more expressed in pericytes, brain endothelial cells and neurons, and significantly less expressed in oligodendrocytes. Broadly, we find evidence that areas associated with cerebrovascular impairment are enriched for expression of genes related to neuron support (pericytes, endothelial cells and perineuronal oligodendrocytes) and neurons themselves. This dichotomy is consistent with the observations from the spatial overlap analysis i.e. cerebrovascular impairment spatially covaries with expression of genes, receptors and processes involved in the coordination of metabolic and vasoreactive responses essential for neuronal homeostasis.

## 4 Discussion

We show that abnormalities in cerebral microvascular function, measured using resting state fluctuation amplitudes, RSFA, persist many months (on average six months in our sample) after hospitalisation for acute COVID-19. The location of these abnormalities in lateral frontal and temporoparietal regions aligns partly with cerebrovascular dysfunction reported in association with ageing (Tsvetanov et al., 2021b, Tsvetanov et al., 2015) preclinical Alzheimer’s disease (Millar et al., 2020a) and systemic cardiovascular health (Tsvetanov et al., 2021b).These post-COVID-19 effects were observed over and above age. These effects are related to severity of the acute illness and the host response in the acute stage. These effects also relate to the post-COVID-19 cognitive function, common indices of mental health, and quality of life at an average of six months after hospitalisation.

In spatial covariance analyses, we found overlap between the regional distribution of this cerebrovascular impairment and spatial distribution of the vasoreactive receptor 5-HT1b and regions with high metabolic demands. 5-HT1b receptor is the dominant contractile 5-HT receptor in cerebral arteries (Barnes and Hoyer, 2021, Nilsson et al., 1999), stimulating vasoconstriction by contracting smooth muscle directly or as a moderator of other vasoconstrictors. Distinct from its effects on vascular tone, the presynaptic 5-HT1b receptor also has an important microvascular anti-inflammatory role, both in the cerebrovascular bed and more generally. Further, its loss has been implicated in progressive cognitive loss and abnormal modulation through descending serotoninergic outputs (Gharishvandi et al., 2020, Heijmans et al., 2021, Mitsikostas et al., 2002, Sibille et al., 2007), which may be relevant for chronic sequelae after COVID-19.

The overlap of cerebrovascular impairment with the regional distribution of aerobic glycolysis and glucose metabolism is spatially concordant with previous reports of hypometabolism in the subacute phase of COVID-19 (Hosp et al., 2021) and neurodegeneration (Vlassenko et al., 2010). This indicates a potential link between cerebrovascular impairment and metabolic dysfunction in frontoparietal regions, which could provide important insights regarding the mechanisms of late neurocognitive dysfunction following COVID-19 infection. Future work should establish whether changes to the microvasculature lead to hypometabolism (Shi et al., 2016) or whether the vulnerability of brain physiology in the chronic phase is due to hypoxic and hypometabolic exposure in the subacute phase (Vestergaard et al., 2020). Collectively, these results demonstrate that the distribution of chronic cerebrovascular impairments related to COVID-19 severity maps to the spatial distribution of processes involved in coordinating metabolic and vasoregulatory responses associated with changes in brain function and cognition.

We also tested whether genes highly expressed in regions with COVID-19-induced cerebrovascular change are preferentially expressed in specific cell types using a molecular atlas of human brain vasculature (Yang et al., 2021). Dominant genes were overexpressed in pericytes, brain endothelial cells and neurons, but underexpressed in oligodendrocytes. The evidence that genes implicated are enriched in pericytes and endothelial cells is particularly interesting. Brain endothelial cells are susceptible to direct SARS-CoV-2 infection through flow-dependent expression of ACE2. The SARS-CoV-2 S protein binding triggers a gene expression profile that may compromise the neurovascular interface (Kaneko et al., 2021). On the abluminal aspect of the neurovascular interface, pericytes express abundantly the angiotensin-converting enzyme-2 (ACE2) receptor (He et al., 2020). The expression can be increased by exposure to the viral S protein, and importantly, potentiated in combination with hypoxia (Khaddaj-Mallat et al., 2021), a mechanism that could account for the modulation of RSFA abnormality by disease severity in our cohort.

Given this biological context the correlation of RSFA abnormalities with disease severity is open to two potential interpretations. One possibility is that these changes in cerebrovascular regulatory integrity are the consequence of direct viral invasion; while the other is that these abnormalities are a consequence of the inflammatory host response, which is a consequence of, but may not scale precisely with, viral infection. Spatial correlation of RSFA abnormality with the expression of ACE2 and Neuropilin-1, or of genes involved in cellular responses to viral infection would have provided supportive evidence of a role for direct viral infection as a mechanism, but we were unable to demonstrate such correlations. These negative findings favour the explanation that host inflammatory responses may be drivers in this context, and merit further investigation as mechanisms of late cerebrovascular regulatory dysfunction, consistent with the hypothesis of long COVID endotheliopathy (Ahamed and Laurence, 2022). It is important to acknowledge that our correlations with regional gene expression are based on expression patterns in normal brain, and that these expression patterns may be substantially altered by the inflammatory milieu that prevails in the context of COVID-19. Consequently, direct examination of peripheral blood gene expression profiling in late COVID-19 survivors would provide additional insights.

Our study has several limitations. We are limited (i) by the relatively small sample size with incomplete data across modalities and patients (Jakobsen et al., 2017), (ii) by sensitivity to domain-specific cognitive impairment (Coen et al., 2016) and (iii) by the absence of longitudinal imaging data in patients and physiological data in controls. We also can not draw any causal inferences from the associations we observe. However, the demonstration of functional microvascular abnormalities following COVID-19 is important to understand the potential mechanisms of persistent cognitive and mental health problems. The association of microvascular abnormalities with late outcomes of relevance to patients, and the fact that they represent an easily accessible biomarker, suggest both a potential therapeutic target and/or a biomarker of treatment effect in interventional studies. It remains to be shown whether the localisation of RSFA abnormalities to regions rich in 5HT-1b receptors is a consequence of overactivity of these receptors (resulting in low cerebral blood flow), underactivity or loss of these receptors (resulting in vasoparalysis and/or inflammation), or a manifestation of flow-metabolism mismatching with inadequate substrate and oxygen delivery. This is relevant as potential therapeutic agents are available to modulate both 5HT-1b function (Barnes et al 2021) and inflammatory response (Group and Sterne, 2021, Zhang et al., 2020).

In summary, we demonstrate that the severity of acute COVID-19 predicts cerebrovascular impairment six months later. The cerebrovascular abnormality was associated with worse cognitive function, mental health, functional recovery, and quality of life months after hospitalisation. Localised across lateral frontotemporoparietal regions, we show that the physiological and genetic signature of this cerebrovascular impairment shapes the composition of cell-types, metabolism and vasoreactivity essential for neuronal homeostasis. Collectively, these results implicate long-lasting COVID-19-related vulnerability of brain systems differentially relying on metabolic physiology and cell biology in support of their functional specialisation.

## CRediT authorship contribution statement

**Kamen A. Tsvetanov:** Conceptualization, Methodology, Software, Formal analysis, Visualization, Writing – original draft, Writing – review & editing. **Lennart R.B. Spindler:** Data curation, Writing – review & editing. **Emmanuel A. Stamatakis:** Writing – review & editing. **Virginia F.J. Newcombe:** Investigation, Writing – review & editing. **Victoria C. Lupson:** Investigation, Writing – review & editing. **Doris A. Chatfield:** Data curation, Investigation, Writing – review & editing. **Anne E. Manktelow:** Data curation, Investigation, Writing – review & editing. **Joanne G. Outtrim:** Data curation, Investigation, Writing – review & editing. **Anne Elmer:** Investigation, Writing – review & editing. **Nathalie Kingston:** Investigation, Writing – review & editing. **John R. Bradley:** Investigation, Writing – review & editing. **Edward T. Bullmore:** Conceptualization, Methodology, Funding acquisition, Project administration, Supervision, Writing – review & editing. **James B. Rowe:** Conceptualization, Methodology, Funding acquisition, Project administration, Supervision, Writing – original draft, Writing – review & editing. **David K. Menon:** Conceptualization, Methodology, Funding acquisition, Project administration, Supervision, Writing – original draft, Writing – review & editing.

## Declaration of Competing Interest

The authors declare that they have no known competing financial interests or personal relationships that could have appeared to influence the work reported in this paper.

## Data Availability

Code and composite data to reproduce manuscript figures and statistical analyses will be made available at https://github.com/kamentsvetanov/covid19_cerebrovascularburden. More detailed data may be available on request, with enquiries directed to dac@bioresource.nihr.ac.uk.
