# Supplementary material for: Hospitalisation for COVID-19 predicts long lasting cerebrovascular impairment: A prospective observational cohort study
Source: Neuroimage Clin. 2022 Nov 7;36:103253. doi: 10.1016/j.nicl.2022.103253 (PMC9639388; doi:10.1016/j.nicl.2022.103253)
Supplement: Supplementary data 1 [file mmc1.docx]

Supplementary Information: Covid-19 severity and cerebrovascular burden

# Supplementary Tables

SI Table 1. Results from commonality analysis using 10.000 permuations indicating that a portion of the variance between COVID-19 Seveirty-LV and RSFA-LV was explained by age (PercentTotal for ‘CV19Severity,Age’), and shared variance between age and cardiorepriratory dysfunction component 1 (CV19Sevitiy,Age,Clinical_PCA1_iAll). COVID-19 Severity remained as the largest unique predictor of RSFA-LV (CV19Severity).
